# Supplementary material for: Design of Anti-Tumor RNA Nanoparticles and Their Inhibitory Effect on Hep3B Liver Cancer
Source: Biomolecules. 2025 Dec 26;16(1):45. doi: 10.3390/biom16010045 (PMC12839093; doi:10.3390/biom16010045)

Figure 1c. Expression of F1B3hT1-RNA nanoparticles in *Escherichia coli*.

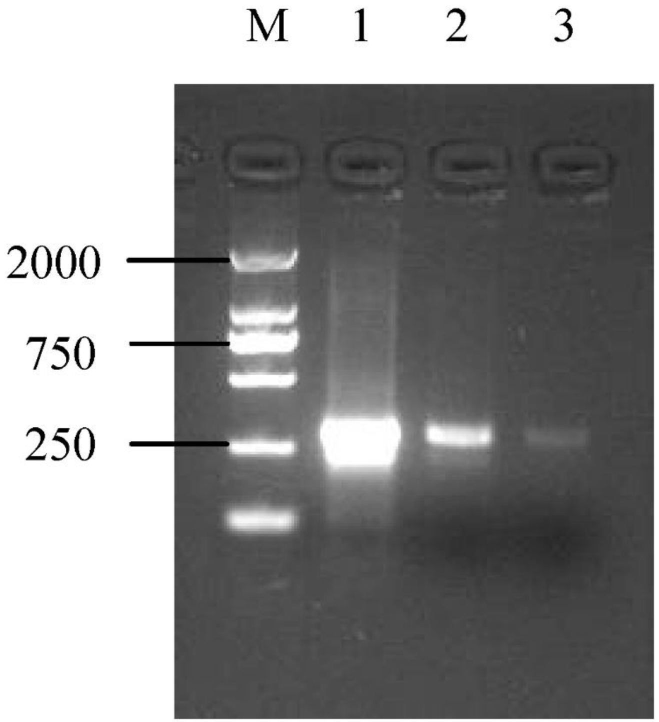

Lane M: DL2,000 DNA Marker

Lane 1: Agarose gel electrophoresis bands of RNA nanoparticles precipitated by LiCl

Lane 2: Agarose gel electrophoresis bands of ten-fold-diluted RNA nanoparticles precipitated with LiCl

Lane 3: Agarose gel electrophoresis bands of twenty-fold-diluted RNA nanoparticles precipitated with LiCl

Figure 5d. Western Blot was used to detect the expression levels of hTERT protein in different treatment groups at 48 h, 72 h, and 96 h.

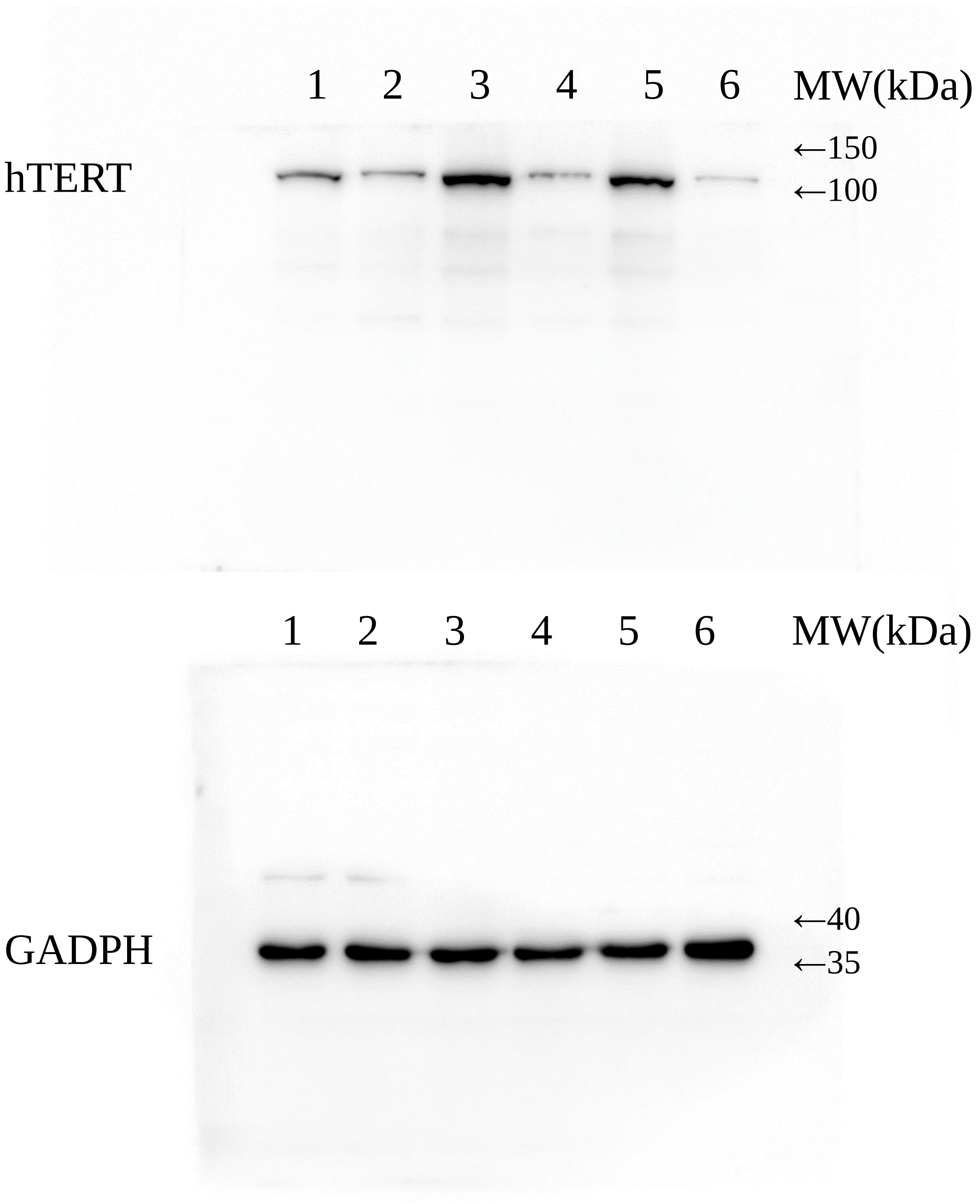

Lane 1: Extraction solution without RNA (48h)

Lane 2: Treated with 150 ng/μl RNA (48h)

Lane 3: Extraction solution without RNA (72h)

Lane 4: Treated with 150 ng/μl RNA (72h)

Lane 5: Extraction solution without RNA (96h)

Lane 6: Treated with 150 ng/μl RNA (96h)

Figure 5e. Western Blot was used to detect the expression levels of BIRC5 protein in different treatment groups at 48 h, 72 h, and 96 h.

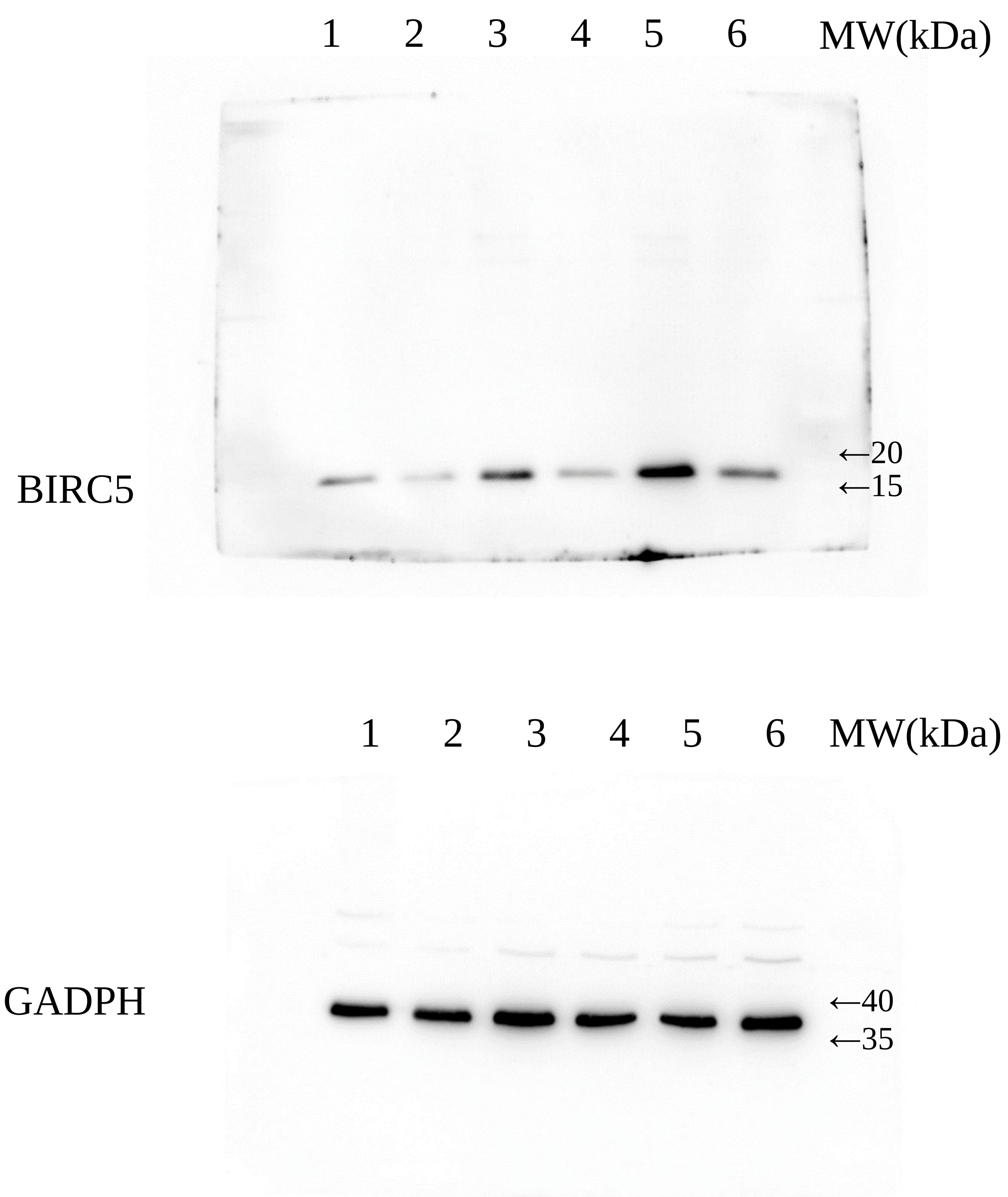

Lane 1: Extraction solution without RNA (48h)

Lane 2: Treated with 150 ng/μl RNA (48h)

Lane 3: Extraction solution without RNA (72h)

Lane 4: Treated with 150 ng/μl RNA (72h)

Lane 5: Extraction solution without RNA (96h)

Lane 6: Treated with 150 ng/μl RNA (96h)

Figure 5f. Western Blot was used to detect the expression levels of FGFR1 protein in different treatment groups at 48 h, 72 h, and 96 h.

Lane 1: Extraction solution without RNA (48h)

Lane 2: Treated with 150 ng/μl RNA (48h)

Lane 3: Extraction solution without RNA (72h)

Lane 4: Treated with 150 ng/μl RNA (72h)

Lane 5: Extraction solution without RNA (96h)

Lane 6: Treated with 150 ng/μl RNA (96h)

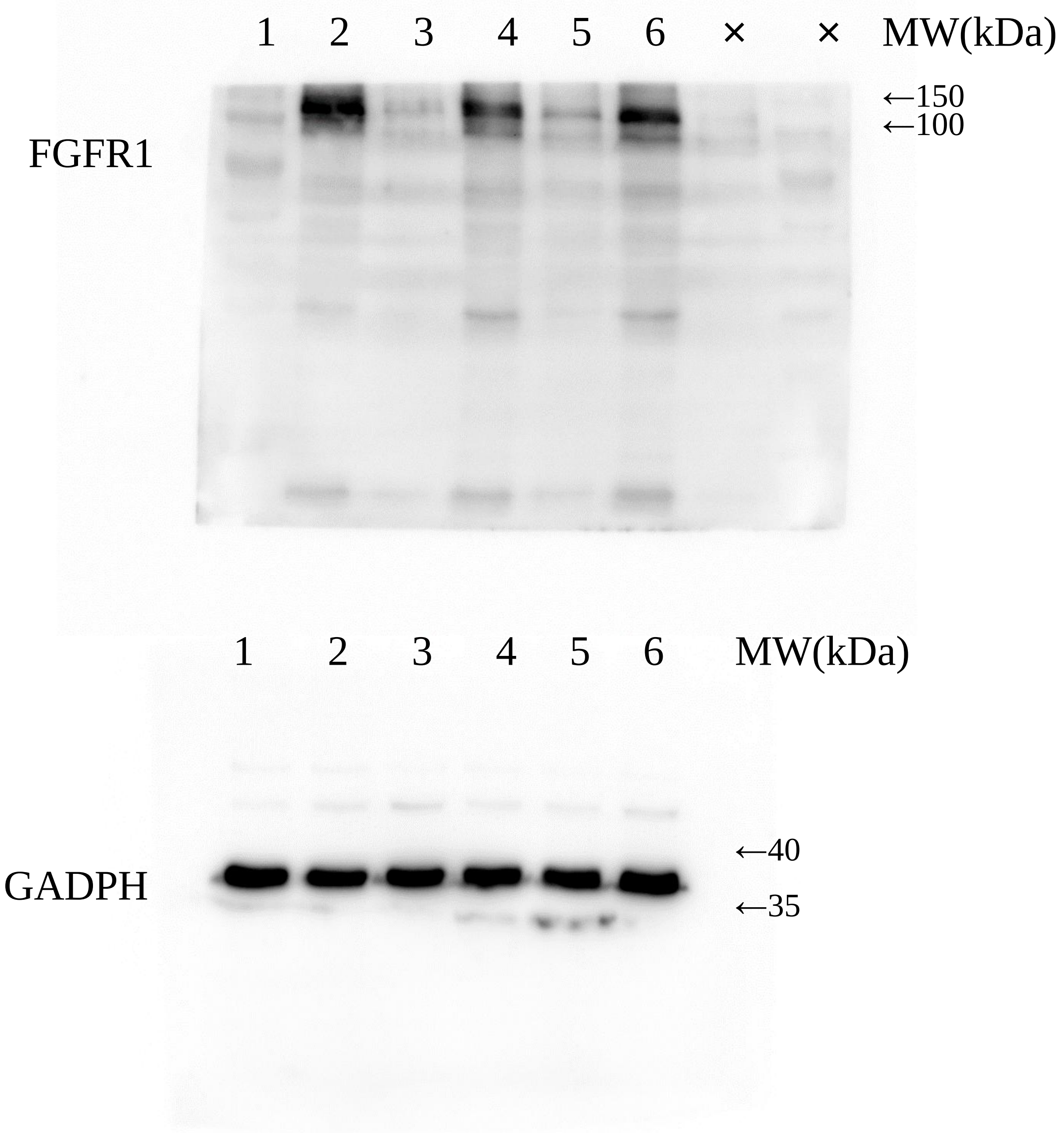

Supplement: Supplementary file 1 [file biomolecules-16-00045-s001.zip › biomolecules-3928646-File S1. Original WB images.pdf]
